# Variation in faecal microbiota in a group of horses managed at pasture over a 12-month period

**DOI:** 10.1038/s41598-018-26930-3

**Published:** 2018-05-31

**Authors:** Shebl E. Salem, Thomas W. Maddox, Adam Berg, Philipp Antczak, Julian M. Ketley, Nicola J. Williams, Debra C. Archer

**Affiliations:** 1 Department of Cellular and Molecular Physiology, Institute of Translational Medicine, University of Liverpool, Liverpool, L69 3BX UK; 2 Department of Surgery, Faculty of Veterinary Medicine, Zagazig University, Zagazig, 44519 Egypt; 3 Department of Musculoskeletal Biology, Institute of Ageing and Chronic Disease, University of Liverpool, Leahurst Campus, Wirral, CH64 7TE UK; 4 Department of Genetics, College of Medicine, Biological Sciences and Psychology, University of Leicester, Leicester, LE1 7RH UK; 5 Centre of Computational Biology and Modelling, Institute of Integrative Biology, University of Liverpool, Liverpool, L69 7ZB UK; 6 Department of Epidemiology and Population Health, Institute of Infection and Global Health, University of Liverpool, Leahurst Campus, Wirral, CH64 7TE UK; 7 Philip Leverhulme Equine Hospital, Institute of Veterinary Science, University of Liverpool, Wirral, CH64 7TE UK

## Abstract

Colic (abdominal pain) is a common cause of mortality in horses. Change in management of horses is associated with increased colic risk and seasonal patterns of increased risk have been identified. Shifts in gut microbiota composition in response to management change have been proposed as one potential underlying mechanism for colic. However, the intestinal microbiota in normal horses and how this varies over different seasons has not previously been investigated. In this study the faecal microbiota composition was studied over 12 months in a population of horses managed at pasture with minimal changes in management. We hypothesised that gut microbiota would be stable in this population over time. Faecal samples were collected every 14 days from 7 horses for 52 weeks and the faecal microbiota was characterised by next-generation sequencing of 16S rRNA genes. The faecal microbiota was dominated by members of the phylum Firmicutes and Bacteroidetes throughout. Season, supplementary forage and ambient weather conditions were significantly associated with change in the faecal microbiota composition. These results provide important baseline information demonstrating physiologic variation in the faecal microbiota of normal horses over a 12-month period without development of colic.

## Introduction

Colic (abdominal pain) is a common cause of death in horses and is a key health concern for horse owners^1^. Multiple epidemiological studies have investigated risk factors for colic and have identified a number of modifiable and non-modifiable risk factors. Knowledge of factors that can be modified allow preventive strategies to be devised to reduce the incidence of colic^2^. Consistently reported factors that increase the likelihood of colic are related to changes in management such as feeding a new batch of forage, a change in type of forage, a change in type and amount of grain-based feed, decreased access to pasture and increased time spent stabled^3–6^. It has been proposed that dietary changes associated with these changes may alter the colonic microflora, inducing changes in colonic pH and volatile fatty acid production, predisposing horses to colic^5,7^.

Colic has been shown to have a seasonal pattern which differs between different populations of horses and between different types of colic^8^. There is a growing body of evidence to suggest that changes in the horse hindgut microbial communities (gut microbiota) in response to diet change^9^, feeding a high-concentrate diet^10,11^, changes in access to pasture^12^ and transportation^13^ may play a role in the relationship between these known risk factors for colic and colic risk. Currently, it is unknown whether stability over time is a primary feature of these microbial populations, particularly under constant management conditions. A single study has investigated stability of faecal microbiota of ponies fed a commercial diet^14^. This study demonstrated that bacterial populations were stable over the two-time periods investigated, but there was significant variation between individuals.

The objectives of the current study were to determine if stability is an integral feature of the horse gut microbiota in a population of normal horses kept solely at pasture over a 52-week period, without other management changes such as stabling and provision of concentrate feed, and to investigate if ambient weather conditions such as rainfall and temperature had any effect on these microbial populations. This would provide important baseline information needed to determine if these changes are different in horses that develop colic.

## Methods

### Animals

The study population consisted of 7 horses that were managed on the same grass paddock year-round (see Supplementary Table S1). The horses were healthy and had no history of change in diet, turnout, transportation, medical conditions nor had any medications been administered in the 30-day period prior to the start of sample collection. None of the horses had a history of colic in the previous 12 months nor had undergone any form of abdominal surgery. Horses had free access to grass and water without administration of concentrate feed or other dietary supplements. During the months of the year when the quality of the grass was insufficient to provide adequate nutrition, horses received supplementary *ad lib* haylage.

### Sample collection

The aim was to investigate changes in the equine gut microbiome over a full 12-month period, to take into account seasonal variation in pasture and ambient weather conditions. Horses were sampled every 14 days between April 2014 and April 2015, contributing 27 sampling points (T1–T27) (see Supplementary Fig. S1). Samples (approximately 200 g each) were collected from the ground immediately following observed defecation from each identified horse and were placed into sealable plastic bags prior to being placed in storage at −80 °C within 2–6 hours of collection. To ensure consistency of the time of the day when samples were collected, the start of each visit to the field was fixed at 0900 hours. Only n = 6 horses contributed samples at all sampling occasions, as one horse was euthanised after T4 due to injury. Samples collected at T15 were excluded as they were mistakenly stored at 4 °C for a week prior to freezing.

### Parasite testing

Horses were tested for gastrointestinal parasite burdens twice during the study. The first faecal worm egg count testing was concurrent with T1 while the second test was concurrent with T21. The results of the worm egg count are provided in Supplementary Table S1. An anthelmintic preparation containing moxedectin and praziquantel (Equest Pramox, Zoetis, UK) had been administered 10 days before T4. To explore the effect of this treatment on the horse faecal microbiota composition, additional faecal samples were collected 3 days post-dosing (samples labelled T4*).

### Meteorological data

Meteorological data were obtained from a local weather station^15^ for each sampling occasion. This station was not functioning during the first 4 sampling occasions and therefore the second nearest weather station to the sampling site was used^16^. The data collected included the lowest, average and highest temperature; rainfall; and maximum and average wind speed.

### DNA extraction, creation of amplicon libraries, sequencing and bioinformatics analysis

DNA was extracted using the QIAamp DNA Stool Mini Kit (Qiagen, UK) according to the manufacturer’s instructions with an additional step of bead beating and initial incubation at 95 °C instead of 70 °C. Amplicon libraries were created by PCR amplification of the V1–V2 variable regions of the bacterial 16S rRNA genes using the universal eubacterial 8F^17^ and 334R^18^ primer set and were submitted for sequencing using the Ion Torrent PGM sequencing technology (Life Technologies, UK). Sequence data were processed using the Quantitative Insights into Microbial Ecology pipeline (QIIME, version 1.9.1)^19^. This included splitting sequences between samples according to their barcode sequences, chimera identification and removal, clustering of sequences open-reference into operational taxonomic units (OTUs) at 97% identity threshold and building an approximately-maximum-likelihood phylogenetic tree. Detailed methods are given in Supplementary Information.

### Data analysis

Statistical analyses were performed in R software environment (version 3.2.5)^20^ using the following add-on statistical packages: ‘phyloseq’ version 1.12.2^21^, ‘ggplot2’ version 1.0.1^22^, ‘nlme’ version 3.1–121^23^, ‘vegan’ version 2.3-0^24^, ‘cluster’ version 2.0.3^25^ and ‘DESeq2’ version 1.8.1^26^.

### Data filtration and normalisation

Alpha diversity analysis was performed on a non-filtered, non-normalised OTU table^27^, whereas other analyses were performed following data filtration and normalisation. Data filtration involved excluding OTUs that were present in <10 samples (approximately 5% of the samples) or were represented by <20 reads from the total sequences. Data filtration has been previously reported to improve diversity analysis^28^ and statistical power to detect differentially abundant OTUs^29^. Data normalisation was implemented by random subsampling (rarefying) to a minimum sequence depth of 9824 reads without replacement to consider unequal sequencing depths among samples.

To explore relative abundance of different bacterial phyla of the horse faecal microbiota, the data were condensed at the phylum taxonomic level and the mean relative abundance of these bacterial groups were presented in an area plot. Samples collected before and following anthelmintic administration (T3 and T4*) were compared for community diversity using the Wilcoxon-signed rank test and for community structure using permutational multivariate analysis of variance (PMANOVA) following calculation of Weighted-UniFrac, Bray–Curtis and Jensen–Shannon divergence dissimilarity metrics. Significant differences in community structure were identified for T4* and therefore these samples were excluded from any downstream analysis.

### Cluster and ordination analysis

The data were clustered using principal coordinates analysis (PCoA) eigenvectors of a Bray–Curtis dissimilarity matrix derived from the normalised OTU table. The most important eigenvectors were chosen and clustered using the partitioning around medoids algorithm (cluster::pam function in R)^30^. The number of clusters (K = 3) was estimated using the gap statistic. Using these 3 clusters, non-metric multidimensional scaling ordination of a Bray–Curtis distance was performed. Weather data were compared between identified clusters using a Kruskal–Wallis rank sum test followed by Dunn’s *post hoc* test for multiple comparison^31^ and Benjamini Hochberg adjustment for false discovery rate^32^. The results of this analysis were presented in boxplots.

Constrained ordination analysis was performed using distance-based redundancy analysis (db-RDA). The model was built using the vegan::ordiR2step function in R. This function performs automatic forward stepwise selection of explanatory variables using the two stopping criteria suggested by Blanchet *et al*.^33^. The function attempts to maximise the adjusted R^2^ at every step, and the procedure stops when the adjusted R^2^ starts to decrease, the adjusted R^2^ of the full model is exceeded, or the selected permutation significance level (*p* = 0.05) is exceeded^24^. Explanatory variables available for model building included: time of sample collection, the highest, average and lowest ambient temperature, highest and average wind speed and rainfall as continuous variables. Season of sample collection, horse identity and type of feed were included as categorical variables. Season of sample collection was classified as spring (March–May), summer (June–August), autumn (September–November) and winter (December–February), while feed was classified as haylage, and grass plus haylage. Variation in the data due to individual horse effect was excluded from model building through the use of the argument ‘Condition’ in the vegan::capscale function in R.

### Alpha diversity analysis

Diversity measures calculated included Chao1 index^34^ for species richness (a measure of the number of species within a community) and Shannon^35^ and Simpson^36^ diversity indices for population diversity (a measure of both species richness and their relative abundance within a community). The change of these estimates over time was first explored using loess regression curves then modelled by linear mixed-effects (LME) modelling procedures. A random intercept and slope model was built for each measure with sampling time included as a fixed effect variable and horse was included as a random effect. The models were built using the maximum likelihood method which allowed exploring the effect of incorporating higher order terms of time into models using Akaike information criterion (AIC)^37^. Higher order terms that resulted in a reduction of AIC by at least 2 were considered significant.

The effect of weather data on alpha diversity measures was also investigated using the LME modelling procedures. Due to high correlation between weather data, six models were built for each diversity measure, each including different combinations of weather data variables. The model that best fitted the data was chosen based on the value of AIC. This was followed by adding other explanatory variables (season of sample collection and type of feed) into the chosen model where the improvement in model fit was tested using AIC. Interaction and higher order terms were also tested in the final model.

### Beta diversity analysis

The community stability over time was investigated by calculating three dissimilarity metrics (Weighted UniFrac, Bray–Curtis, and Jensen–Shannon divergence) as sample to sample distances (distances between consecutive time points) within each horse^30^. The trend of change over time was first explored using loess regression curves followed by fitting random intercept and slope LME models. Within each model the sampling time points were included as a fixed-effect variable and the horse as a random effect. The number of higher order terms that best fitted the data was identified by comparing models with different higher order terms using AIC. Predication plots from final models with 95% confidence limits were created. The effect of weather data on calculated distances was also investigated as was described for the alpha diversity measures.

### Differential abundance analysis

Differentially abundant OTUs that were associated with feed, highest temperature, rainfall and season (these variables were selected based on the results of cluster and diversity analysis) were identified using negative binomial (NB) models. The models were built using the DESeq. 2::DESeq function in R. Before fitting models, the data were further filtered to remove OTUs that were present in less than 25% of the samples. Variance stabilising transformation of the data was executed as a part of model fitting and therefore the data were not rarefied in advance. A separate model was run for each of these variables and the number of differentially abundant OTUs (adjusted *p* of < 0.01) were arranged in a Venn diagram.

### Data availability Statement

The datasets generated during and/or analysed during the current study are available from https://figshare.com/s/ee85a3e1fdd5c68ed746.

### Ethics approval and consent to participate

The study was approved by the University of Liverpool Veterinary Research Ethics Committee (VREC207) and informed consent was obtained from the owner of the horses. All experiments were performed in accordance with relevant guidelines and regulations.

## Results

A total of 166 faecal samples were collected during the study. These generated 4,304,569 high quality, non-chimeric sequences that were annotated to 58,544 OTUs. The distribution of the number of reads across samples was as follows: a minimum of 10,220 reads, a maximum of 62,200 reads, a median of 24,180 reads, and an interquartile range of 20,830–29,960 reads. Filtration of low abundant taxa resulted in exclusion of 201,757 sequences (4.7% of the original total read count) and 46,211 OTUs, leaving 12,333 OTUs available for further downstream analyses.

**Figure 1:**
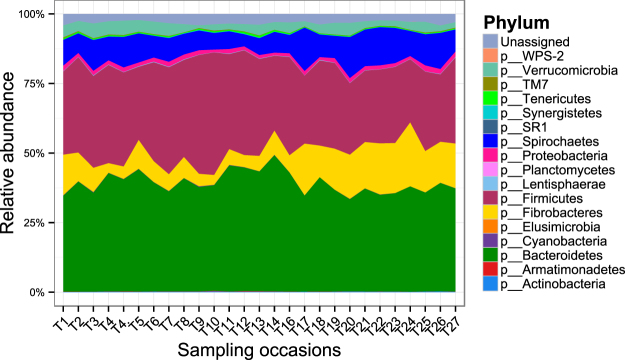
An area plot of relative abundance of different bacterial phyla identified in the data.

The relative abundance of different bacterial groups, condensed to the phylum taxonomic level, is shown in Fig. 1 and Supplementary Table S2. The communities were dominated by members of the Firmicutes and Bacteroidetes phyla with a total of 6 bacterial phyla identified at a relative abundance of ≥1%. These included Bacteroidetes, Firmicutes, Fibrobacteres, Spirochaetes, Verrucomicrobia and Proteobacteria. Taxa that belonged to an unknown phylum represented 2.4% of the communities. A prominent pattern of change of relative abundance of different bacterial phyla was identified (Fig. 1). Some bacterial phyla such as Bacteroidetes and Proteobacteria showed relatively stable relative abundance throughout the study period, whereas others including Firmicutes, Fibrobacteres, Spirochaetes and Verrucomicrobia showed a biphasic change over the 12 months studied. The increase in relative abundance of Fibrobacteres and Spirochaetes was associated with a decrease in relative abundance of Firmicutes and Verrucomicrobia and vice versa. This pattern of change in relative abundance over time was consistent among all horses included in the study (see Supplementary Fig. S2). A total of 27 bacterial classes were identified in the data based on the RDP classifier^38^. Classes that were present at ≥1% included Bacteroidia, Clostridia, Fibrobacteria, Spirochaetes, Verruco-5, Erysipelotrichi and Alphaproteobacteria. Of all OTUs identified in the dataset, 81.23% and 96.1% were not assigned to a genus or a species taxonomic level, respectively.

### Cluster and ordination analysis

**Figure 2:**
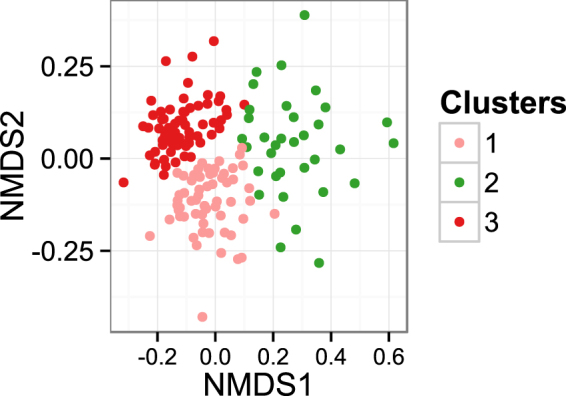
Non-metric multidimensional scaling of a Bray–Curtis dissimilarity matrix derived from the normalised OTU table. The sampling time points were coloured by clusters identified in the data.

**Figure 3:**
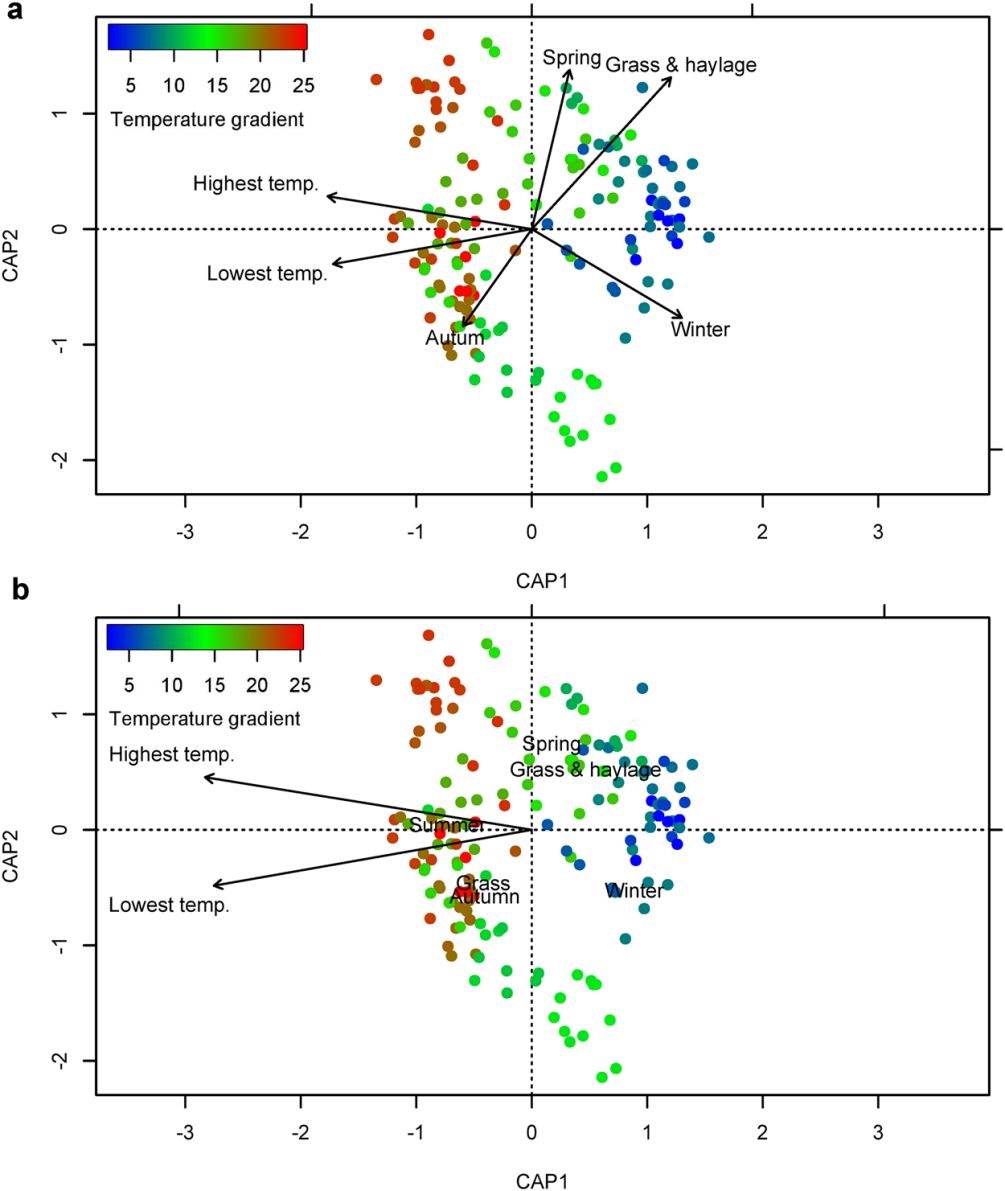
Ordination plots of the first two axes from the distance-based redundancy analysis model. The figure displays biplot scores of constraining variables (coordinates of the tips of the vectors representing the explanatory variables) (**a**) and centroids of factor constraints (coordinates of categories of factor variables) (**b**). The dots represent samples collected during the study period.

The gap statistic suggested that there were 3 clusters in the data. Ordination analysis using non-metric multidimensional scaling of a Bray–Curtis dissimilarity matrix derived from the OTU table is shown in Fig. 2. Weather data apart from rainfall differed significantly among these clusters (see Supplementary Fig. S3). Variables retained in the final model of db-RDA included season of sample collection, type of feed (grass vs. grass &haylage), and the lowest and highest ambient temperature (Fig. 3). The model explained 13.2% of variation in the data (adjusted R^2^ = 0.13).

### Alpha diversity analysis

A non-linear trend of change over time was prominent in exploratory loess regression plots (see Supplementary Fig. S4). Testing of different higher order terms of time within the LME models showed that a significant non-linear trend (cubic polynomial) was evident only for the Shannon diversity index (see Supplementary Fig. S5). Table 1 presents the results of LME modelling of three diversity measures. Of the environmental variables tested, only ambient temperature was significantly associated with the alpha diversity measures.

**Table 1:** Results of linear mixed-effects modelling of alpha diversity measures.

Variable	Value	Std. error	DF	t-value	*p* value
*Regression of alpha diversity measures against time:*					
**Chao1 index**					
Intercept	8250.9	395.85	152	20.84	2.04 × 10^−46^
Time	108.36	20.75	152	5.22	5.74 × 10^−7^
**Shannon diversity index**					
Intercept	6.92	0.09	150	76.99	1.76 10^−122^
Time	0.33	0.63	150	0.52	0.61
Time^2^	−1.01	0.42	150	−2.39	0.02
Time^3^	1.098	0.63	150	1.73	0.085
**Simpson diversity index**					
Intercept	0.99	0.00	152	420.23	6.80 × 10^−235^
Time	0.0001	0.00	152	0.93	0.36
*Regression of alpha diversity measures against other explanatory variables:*					
**Chao1 index**					
Intercept	11236.68	406.56	152	27.64	3.71 × 10^−61^
Highest temperature	−101.275	23.67	152	−4.28	3.32 × 10^−5^
**Shannon diversity index**					
Intercept	6.72	0.11	152	63.43	2.89 × 10^−111^
Lowest temperature	0.02	0.007	152	3.22	0.002

### Beta diversity analysis

**Figure 4:**
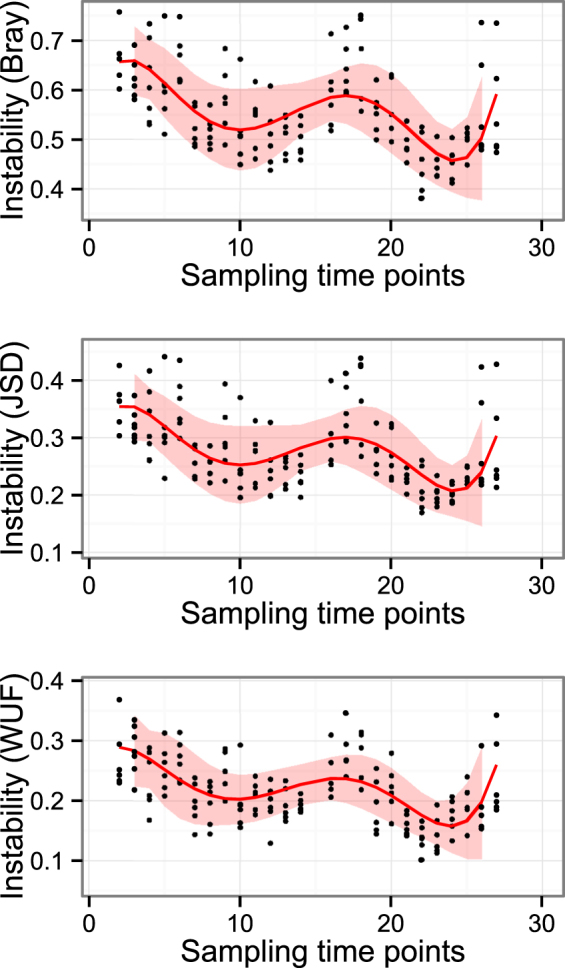
Regression of distances between consecutive sampling time points against time. Red lines are the regression lines from the linear mixed-effects models and the shades are the 95% confidence limits of the prediction. The time was included as a fifth-degree polynomial term.

Distances between consecutive sampling time points showed a clear non-linear trend of change over time on exploratory plots (see Supplementary Fig. S6). Modelling these distances using LME modelling showed that the trend of change was best described with a fifth-degree polynomial term of sampling time. Prediction plots from these models are given in Fig. 4. The association between various environmental variables and calculated distances was also investigated using LME models and results of final models are presented in Table 2. Highest ambient temperature and rainfall were significantly associated with the three dissimilarity measures calculated from the data.

**Table 2:** Results of linear mixed-effects modelling of beta diversity measures.

Variable	Value	Std. error	DF	t-value	*p-*value
*LME modelling of beta-diversity measurements against time:*					
**Bray–Curtis**					
Intercept	0.56	0.01	141	90.63	7.17 × 10^−127^
Time	−0.45	0.09	141	−5.30	4.35 × 10^−7^
Time^2^	0.17	0.10	141	1.66	0.1
Time^3^	−0.18	0.01	141	−1.81	0.07
Time^4^	0.28	0.10	141	2.70	0.008
Time^5^	0.37	0.08	141	4.82	3.69 × 10^−6^
**Jensen–Shannon**					
Intercept	0.28	0.005	141	57.78	4.74 × 10^−100^
Time	−0.33	0.06	141	−5.51	1.64 × 10^−07^
Time^2^	0.13	0.08	141	1.64	0.10
Time^3^	−0.13	0.08	141	−1.58	0.12
Time^4^	0.20	0.08	141	2.38	0.02
Time^5^	0.26	0.06	141	4.13	6.19 × 10^−5^
**Weighted UniFrac**					
Intercept	0.22	0.006	141	38.42	1.42 × 10^−76^
Time	−0.28	0.06	141	−4.63	8.35 × 10^−6^
Time^2^	0.13	0.08	141	1.65	0.10
Time^3^	−0.06	0.05	141	−1.20	0.23
Time^4^	0.22	0.08	141	2.66	0.01
Time^5^	0.20	0.06	141	3.57	4.8 × 10^−4^
*Regression of beta diversity measures against other explanatory variables:*					
**Bray–Curtis**					
Intercept	0.48	0.02	144	27.08	2.30 × 10^−58^
Highest temperature	0.004	0.001	144	4.12	6.42 × 10^−5^
Rainfall	0.01	0.004	144	3.62	4.07 × 10^−4^
**Jensen–Shannon divergence**					
Intercept	0.22	0.013	144	16.39	9.88 × 10^−35^
Highest temperature	0.003	0.001	144	4.04	8.78 × 10^−5^
Rainfall	0.009	0.003	144	3.4	8.84 × 10^−4^
**Weighted UniFrac**					
Intercept	0.17	0.01	144	13.62	1.16 × 10^−27^
Highest temperature	0.003	0.001	144	3.73	2.75 × 10^−4^
Rainfall	0.008	0.002	144	3.06	0.003

As a measure of stability of the community over the course of sample collection, 3 dissimilarity metrics were measured between consecutive sampling time points within each horse and regressed against time and environmental variables.

### Differential abundance analysis

**Figure 5:**
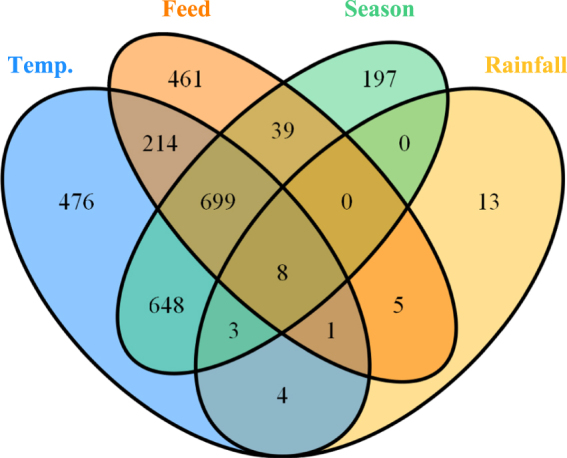
A Venn diagram showing the number of differentially abundant OTUs significantly associated with each of tested variables.

The number of OTUs associated with each of the variables tested in NB models are given in Fig. 5. Large number of these OTUs were shared between the tested variables which indicated high correlation between these variables.

## Discussion

This work is novel in being the first study to investigate the faecal microbiota of horses over a prolonged period enabling seasonal effects to be studied. This work is important in providing baseline information about the normal horse faecal microbiota. This information is essential prior to exploring differences between the faecal microbiota in normal horses under conditions of minimal management and those managed more intensively (e.g. concentrate feed and stabling) or with horses that have developed or are at high risk of colic. This work demonstrates that the faecal microbiota is highly dynamic and responds distinctly to dietary factors and ambient environmental conditions in normal horses. Furthermore, whilst a clear pattern of change in the faecal microbiota was evident over the 12 months under investigation, this did not result in any clinical abnormalities (including colic), developing in the study horses. These findings suggest that any proposed relationship between change in management and altered risk of colic that solely considers shifts in gut microbiota composition is too simplistic and merits more detailed investigation.

The faecal microbiota of the study horses was found to be in a continuous process of adaptation and change in association with alterations in grass, supplementary forage and ambient weather conditions. Several studies have reported changes in the faecal microbiota of horses receiving different diets^39,40^, which is consistent with the findings of the current study. Dynamic adaptation of the gut microbiota has been observed in people^41^ and similar seasonal shifts in gut microbiota composition in response to seasonal dietary variation have also been reported for other animal species^42,43^. Based on these results, it is plausible that intestinal dysfunction/colic could develop either because of lack of adaptation of gut microbiota in some horses or due to major shifts/disruption in gut microbiota composition as a response to marked, sudden management changes^11^.

In the current study, it was not possible to determine how quickly the gut microbiota may adapt to a change in diet because of the duration of time between the sampling occasions and the gradual change in diet (e.g. natural gradual decrease in the amount of grass available to the horses under investigation). It was not possible to perform more frequent sampling due to financial constraints and this is an area for future research. Previous studies have reported that the composition of the horse gut microbiota can shift in response to new diets within 4–6 days^9,12^. It has been shown that dietary variations among animal species including humans and horses is not only associated with compositional differences in gut microbiota, but also functional differences i.e. there is a strong relationship between the microbial community composition and function^44^. Compositional adaption of the gut microbiota has been reported to be rapid; however, functional adaptation may take longer. A study that investigated the fermentation capacity of the pig gut microbiota following a dietary shift reported that a 19-day period was not enough to achieve a full adaptation to a different diet^45^. Similar studies in horses, however, are lacking and this is an area that merits further investigation.

The results of the current study suggest that fluctuations in the composition of the horse faecal microbiota associated with weather and dietary (forage) variations is normal, although only a small number of normal horses were studied. Several epidemiological studies in horses have reported increased risk of colic associated with changes in feeding practices^3,5,7,46^. Both the current study and previous horse microbiota studies reported significant inter-horse variation in gut microbiota composition^14,40,47^, which may partially explain the variation in the propensity of some horses to develop colic. Further work is required in this area including more frequent sampling in horses undergoing sudden changes in diet to further identify changes in the faecal microbiota that may occur and how these differ in a larger number of horses, including horses at greater risk of colic.

The influence of weather (ambient temperature and rainfall) on the horse faecal microbiota identified in the current study is interesting. This effect could be either due to direct correlation between weather conditions and feed types available for the horses, or because of the effect of weather on the composition of environmental bacteria (soil and grass/haylage microbiota). Alterations in the composition of soil microbial populations have been associated with changes in weather conditions. A study that investigated the effect of rainfall on grassland soil microbial communities reported a significant influence of soil moisture and temperature on the composition of associated microbial populations^48^. Environmental bacteria ingested with feed can survive enzymatic digestion in the stomach and small intestine and can colonise the hindgut resulting in shifts in gut microbiota. This was confirmed in a recent human study^49^ where foodborne microbes were identified in faecal microbiota. This is also an area for further investigations in horse microbiota studies.

The most prominent change in the horse gut microbiota identified in the current study was the increased relative abundance of members of the phylum Fibrobacters and Spirochaetes when haylage was introduced into the horses’ diet. This increase appeared to be associated with a decrease in the relative abundance of members of the phylum Firmicutes. Fibrobacteres was the third most abundant phylum in the current study with a relative abundance that ranged from 3.45% to 23% across the study period which is in agreement with previous horse microbiota studies^50–52^. The members of the phylum Fibrobacteres are defined as anaerobic, Gram-negative, non-spore forming, cellulolytic, non-motile rods. It includes a single genus (*Fibrobacter*), previously classified under the genus *Bacteroides*, with only two culture representatives including: *F. succinogenes* and *F. intestinalis*^53^. This bacterium is known for its efficacy in hydrolysing plant cellulose^54^, which may explain its increase in association with introduction of haylage into the horses’ diet.

The horse faecal microbiota was dominated by members of the Firmicutes and Bacteroidetes phyla in the current study. This finding is consistent with multiple studies that used 454-Pyrosequencing technology to sequence 2 or 3 hypervariable regions of the bacterial 16S rRNA genes from faecal samples^9,39,40^. A recent series of studies that characterised the horse faecal microbiota reported that the communities were dominated by members of Firmicutes and Verrucomicrobia phyla^52,55^. The latter studies originated from the same institution and used the Illumina MiSeq sequencing technology to sequence a single hypervariable region of the bacterial 16S rRNA genes. Variation in the results obtained between sequencing platforms, number and the type of 16S rRNA hypervariable regions used have been widely reported^56–59^ and could explain the differences between the studies. In the current study, the V1–V2 variable regions were chosen based on previous work investigated the horse faecal microbiota^39,50^ and prior experience working on these regions in the authors’ laboratory. Furthermore, 2–6 hours’ storage of faecal samples at room temperature prior to freezing in the current study was unlikely to affect the results obtained^60^.

There was an obvious lack of resolution of sequence data generated in the current study, as a large proportion of OTUs were not identified at genus or species taxonomic levels. It is unknown however, if this was associated with any technical errors or with the type of the NGS technology used. Similar observations were described in a study that used the 454 Pyrosequencing technology to characterise the faecal microbiota of a group of Thoroughbred horses and it was suggested that this may be due the presence of numerous previously uncharacterised bacteria in the horse gut^40^. The horses in the present study also consisted of a variety of ages and breeds and underwent other management changes such as administration of anthelmintics. However, these horses and their management were reflective of the way a broad number of horses are managed in the UK and provides a ‘baseline’ regarding the faecal microbiota composition in this population of horses over a 12-month period.

In conclusion, the current study has provided important baseline information about variation of the faecal microbiota in a group of horses managed non-intensively at grass over a 52-week period. These findings suggest that changes in the faecal microbiota are a normal adaptation to dietary and environmental changes in managed horses. More research is required to investigate how the equine microbial community composition and function change in response to other more intensive management changes and whether colic is related to associated individual differences in disturbance of the gut microbiota.

## Electronic supplementary material

Supplementary information
