# Supplementary material for: Variation in faecal microbiota in a group of horses managed at pasture over a 12-month period
Source: Sci Rep. 2018 May 31;8:8510. doi: 10.1038/s41598-018-26930-3 (PMC5981443; doi:10.1038/s41598-018-26930-3)
Supplement: Supplementary file 1 — Supplementary information [file 41598_2018_26930_MOESM1_ESM.pdf]

Variation in faecal microbiota in a group of horses managed at pasture over a 12-months period

Shebl E. Salem[1,2*], Thomas W. Maddox[3], Adam Berg[4], Philipp Antczak[5], Julian M. Ketley[4], Nicola J. Williams[6], Debra C. Archer[6,7]

[1]*Department of Cellular and Molecular Physiology, Institute of Translational Medicine, University of Liverpool, Liverpool L69 3BX, UK.* s.e.shebl@liverpool.ac.uk, s.e.shebl@gmail.com

[2]*Department of Surgery, Faculty of Veterinary Medicine, Zagazig University, Zagazig 44519, Egypt.*

[3]*Department of Musculoskeletal Biology, Institute of Ageing and Chronic Disease, University of Liverpool, Leahurst Campus, Wirral CH64 7TE, UK.*

[4]*Department of Genetics, College of Medicine, Biological Sciences and Psychology, University of Leicester, Leicester LE1 7RH, UK*

[5]*Centre of Computational Biology and Modelling, Institute of Integrative Biology, University of Liverpool, Liverpool L69 7ZB, UK*

[6]*Department of Epidemiology and Population Health, Institute of Infection and Global Health, University of Liverpool, Leahurst Campus, Wirral CH64 7TE, UK.*

[7]*Philip Leverhulme Equine Hospital, Institute of Veterinary Science, University of Liverpool, Wirral CH64 7TE, UK.*

Supplementary Methods

DNA extraction

Frozen faecal samples were thawed at room temperature overnight and were homogenised using a sterile spatula prior to DNA extraction. The DNA was extracted from approximately 200 mg of faecal material per sample using the QIAamp DNA Stool Mini Kit (Qiagen, UK) according to the manufacturer's instructions. To maximise bacterial cell lysis, samples were subjected to bead beating after the addition of the InhibitEX buffer using a sterile 3 mm tungsten carbide beads in a TissueLyser II (Qiagen, UK) at a frequency of 30 Hz for 2 minutes. Samples were also initially incubated at 95°C instead of 70°C for the same purpose. Successful DNA extraction was verified by gel electrophoresis. Extracted DNA was stored at –20°C until needed. The median time between sample collection and DNA extraction was 109 days (range 47–184 days; interquartile range 83, 136.5 days).

Creation of amplicon libraries and sequencing

The universal eubacterial 8F[1] and 334R[2] forward and reverse primer set were used to amplify the V1–V2 hypervariable regions of the bacterial 16S rRNA gene. The forward primer (5'-**CCATCTCATCCCTGCGTGTCTCCGACTCAG** | BarcodeX | Barcode Adapter | *AGAGTTTGATCCTGGCTCAG*-3') consisted of the 30 nucleotide Ion adapter 'A' sequence (sequence in bold), 10–12 nucleotide sample-specific oligonucleotide barcode sequence (Ion Xpress barcodes, Life Technologies, UK), 3 nucleotide linker sequence and the 20 nucleotide 8F primer (sequence in italic), while the reverse primer (5'-**CCACTACGCCTCCGCTTTCCTCTCTATGGGCAGTCGGTGAT** | *TGCCTCCCGTAGGAGTCTG* -3') consisted of the Ion 'P1' sequence (sequence in bold) and the 19 nucleotide 334R primer (sequence in italic).

Samples were amplified in triplicates of 50 μl of polymerase chain reaction (PCR) mixtures[3]. Each PCR reaction mixture consisted of 10 μl of genomic DNA initially diluted 1:10 in nuclease-free water (Sigma-Aldrich, UK), 10 μl 5X Q5 reaction buffer, 10 μl 5X Q5 high GC enhancer, 200 μM dNTPs, 0.5 μM of each of the forward and reverse primers, one unit Q5® High-Fidelity DNA polymerase (New England Biolabs, UK) and nuclease free water up to 50μl. Thermal cycling conditions in T100 Thermal Cycler (Bio-Rad, UK) consisted of initial denaturation at 98ºC for 5 min followed by 25 cycles of denaturation (98 ºC for 20 sec), annealing (53 ºC for 30 sec) and extension (72 ºC for 40 second), then a final extension at 72 ºC for 5 min. Triplicate PCR products were pooled and successful amplification was verified by gel electrophoresis.

The DNA was quantified by resolving 5μl from each of the amplification pools on 1.5% agarose in 1X TAE buffer gel stained with ethidium bromide and their fluorescence intensities were compared to those of known molecular weight

standards (HyperLadder 1kb marker, Bioline, UK) using the GeneTools analysis software (Syngene, UK). Equimolar ratios of a maximum of 56 samples were then mixed to create 3 amplicon mixtures. Amplicon mixtures were purified using the E.Z.N.A.® Cycle-Pure Kit (OMEGA bio-tek, USA) according to the manufacturer's instructions. Purified amplicon mixtures were subjected to gel electrophoresis and the desired bands were excised on a blue-light transilluminator. Retrieval of amplicons from the agarose gel matrix was achieved via spin columns developed at the University of Leicester. Apertures of approximately 1 mm were introduced into the base of 0.5 ml microcentrifuge tubes, packed to the transition of the conical section with polymer filtration wool. Adapted 0.5 ml tubes were then mounted inside 1.5 ml microcentrifuge tubes and excised gel fragments were loaded above the filtration wool. Tubes were twice subjected to centrifugation at 9000 × g for 2 minutes, providing eluates for purification with the E.Z.N.A.® Cycle-Pure Kit. Pooled amplicon mixtures were then subjected to final quality control on the Agilent 2100 BioAnalyser (Agilent Technologies, USA) according to the manufacturer's instructions and submitted for sequencing using the Ion Torrent PGM sequencing technology (Life Technologies, UK).

Sequence filtering, OTU clustering and chimera removal

The data generated from sequencing of 16S rRNA gene amplicons were processed using the Quantitative Insights into Microbial Ecology pipeline (QIIME, version 1.9.1)[4]. Sequences from different samples were demultiplexed according to their barcode sequences using the 'split_libraries.py' QIIME script. Quality filtration parameters of this step included a minimum quality score of 20, truncation of low quality sequences at the first base if a 20 bp sliding window was found, a maximum barcode error of zero, a maximum primer mismatch of 2, a maximum length of homopolymer of 5, a maximum number of ambiguous bases of 2, a minimum and a maximum read length of 200 bp and 500 bp, respectively. From a total of 14,724,893 input sequences, this filtration step yielded 4,741,263 quality sequences (32.2 % of the initial sequence count). Chimeric sequences were identified using the UCHIME algorithm[5] informed by the Greengenes (version 13.8) reference database[6] and removed from the data.

Sequences were then clustered open-reference into operational taxonomic units (OTUs) at 97% identity threshold using USEARCH (version 6.1.544)[7]. A representative sequence from each OTU cluster was aligned to Greengenes core set (version 13.8) [6] using PyNast[8]. Taxonomic assignments of OTU representatives were made using the Ribosomal Database Project (RDP) classifier (version 2.2)[9] informed with the Greengenes reference database at 80% confidence limit. Representative sequences were then filtered to remove gaps and hypervariable regions using Lane mask before creating an approximately-maximum-likelihood phylogenetic tree using FastTree[10].

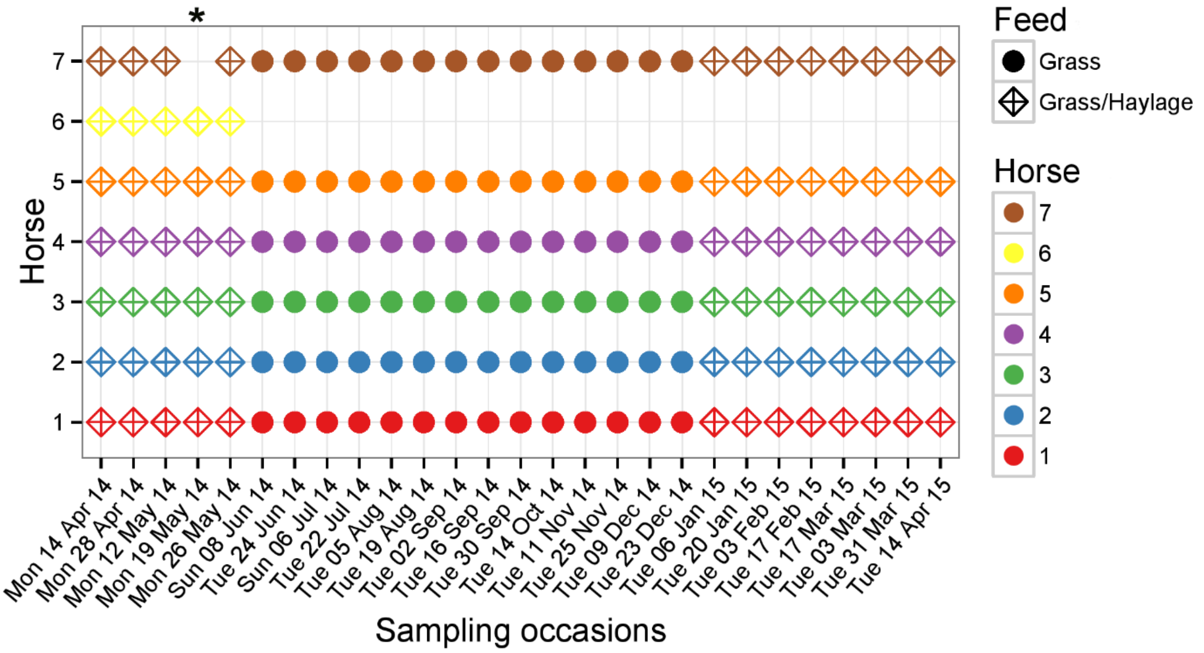

Supplementary Figure S1. A schematic diagram of the sampling strategy used in the study. Samples marked with an asterisk were collected to investigate the effect of anthelmintic treatment (given three days earlier to this sampling date) on the horse faecal microbiota.

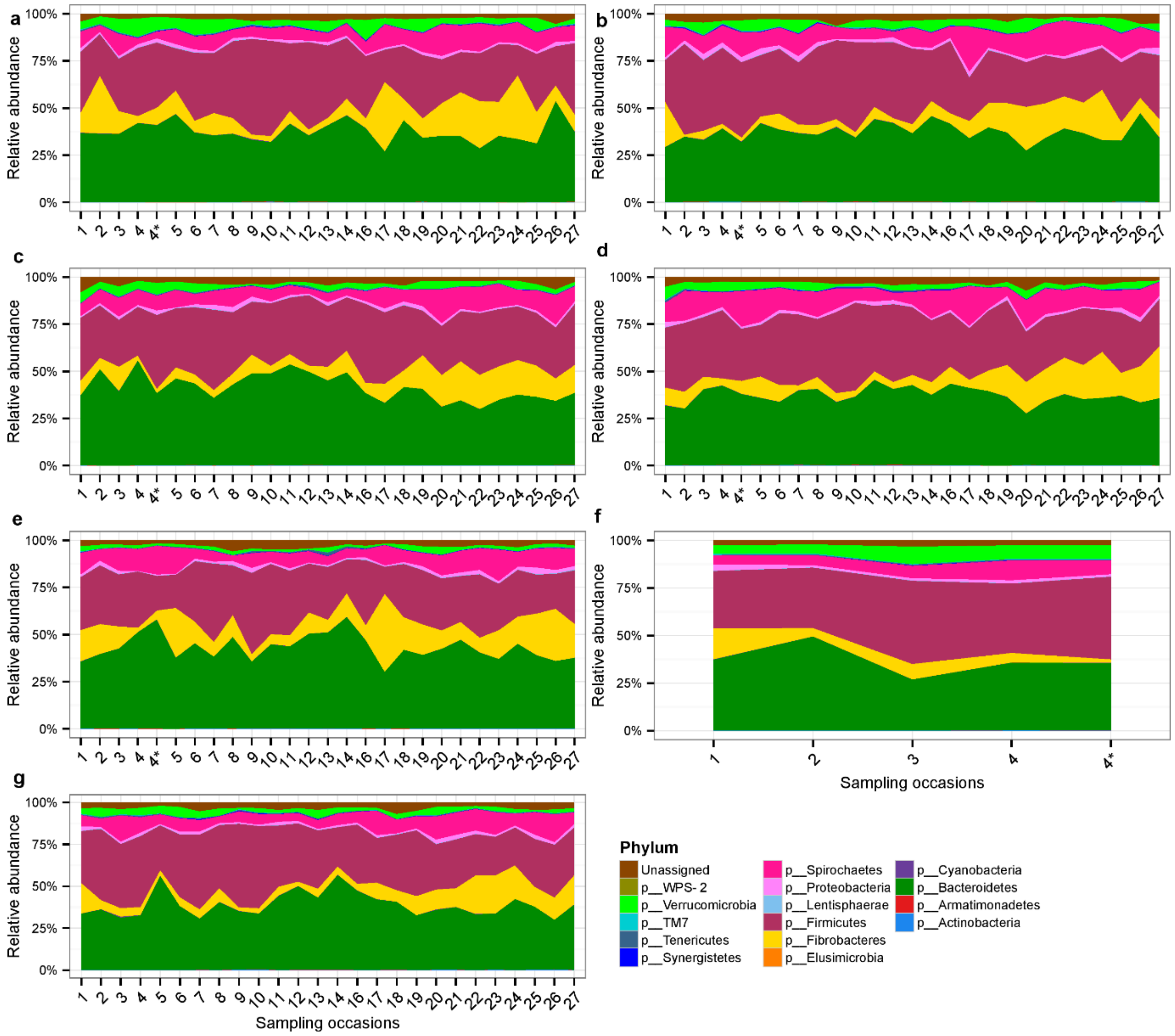

Supplementary Figure S2. Area plots of relative abundance of different bacterial phyla identified in faecal microbiota of each of the horses included in the study. Graph panels a-g represent horses 1-7.

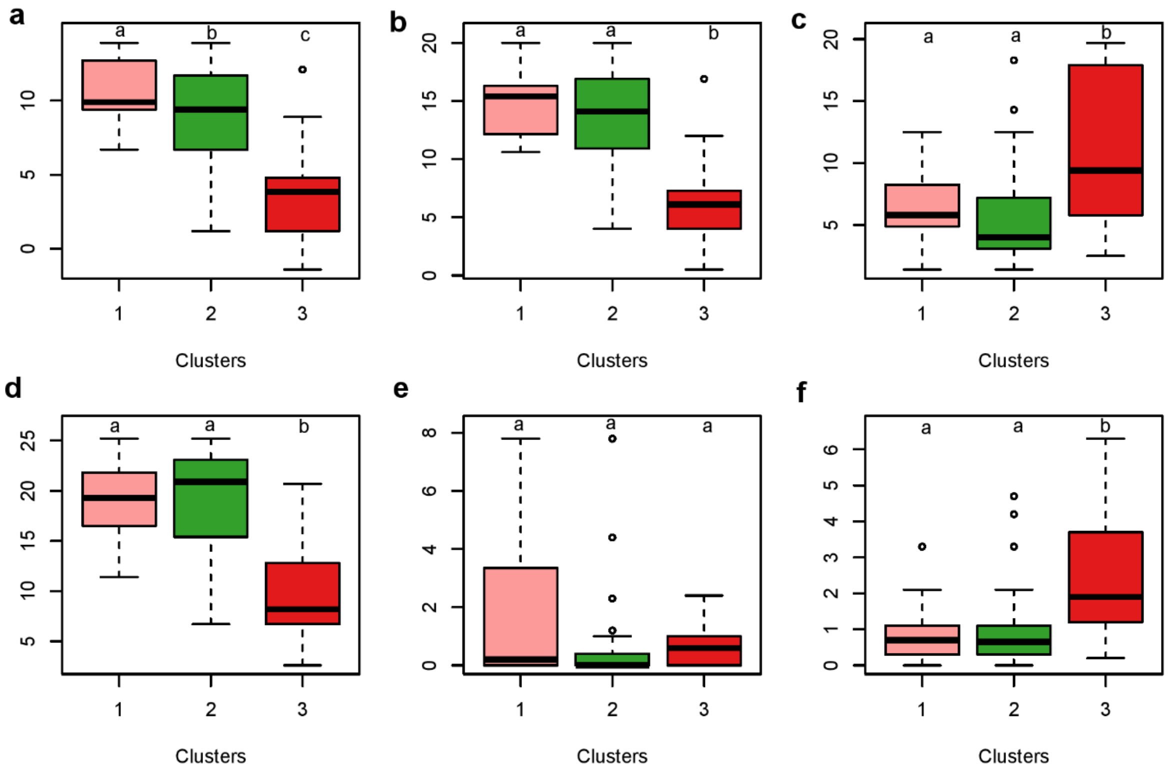

Supplementary Figure S3. Boxplots comparing weather data variables among the three clusters identified in the data. Weather data included lowest (a) and average (b) temperature, highest wind speed (c), highest temperature (d), rainfall in mm/h (e), and average wind speed (f). Clusters with different letters are significantly different at an adjusted $p<0.05$.

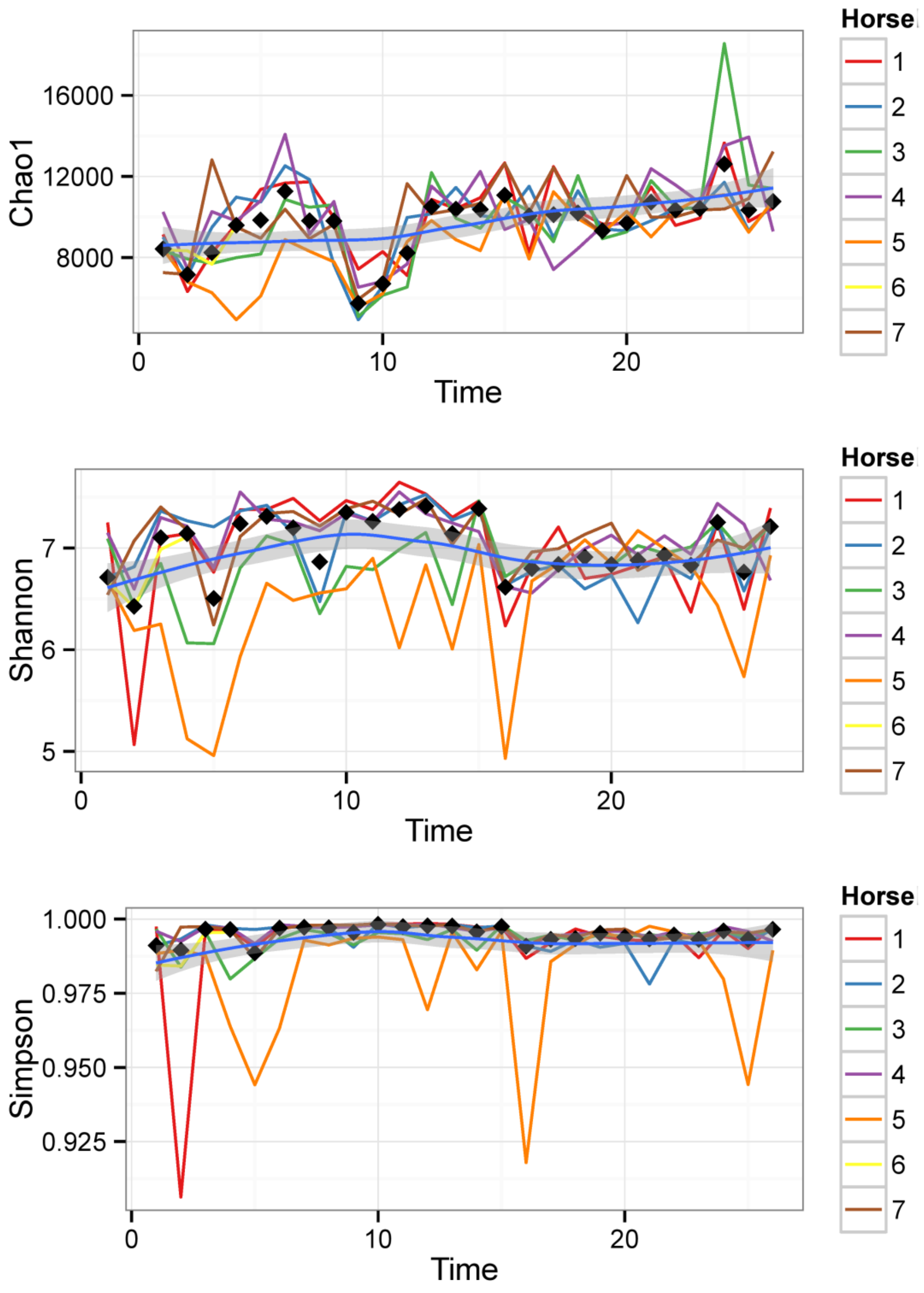

Supplementary Figure S4. Empirical growth plots of alpha diversity measures (Chao1 index [a], Shannon index [b], Simpson index [c]) superimposed with a loess regression line. Black diamonds are the median values at each of the sampling occasions.

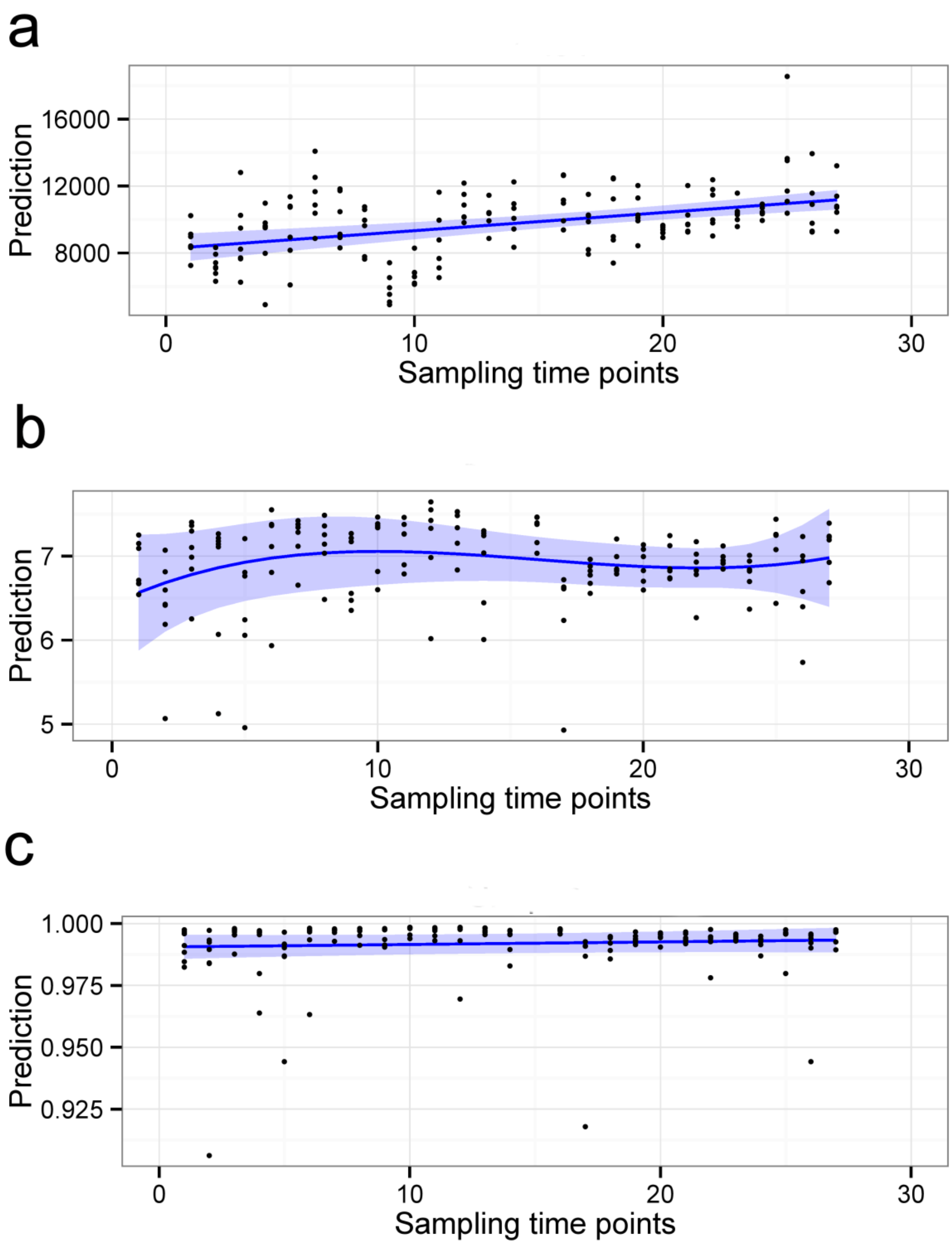

Supplementary Figure S5. Plots of alpha diversity measures (Chao1 [a], Shannon [b], and Simpson [c]) against sampling time points. Blue lines are the regression lines from the linear mixed-effects models and the shades are the 95% confidence limits of the prediction. The models included time as a fixed effect variable and horse as a random effect.

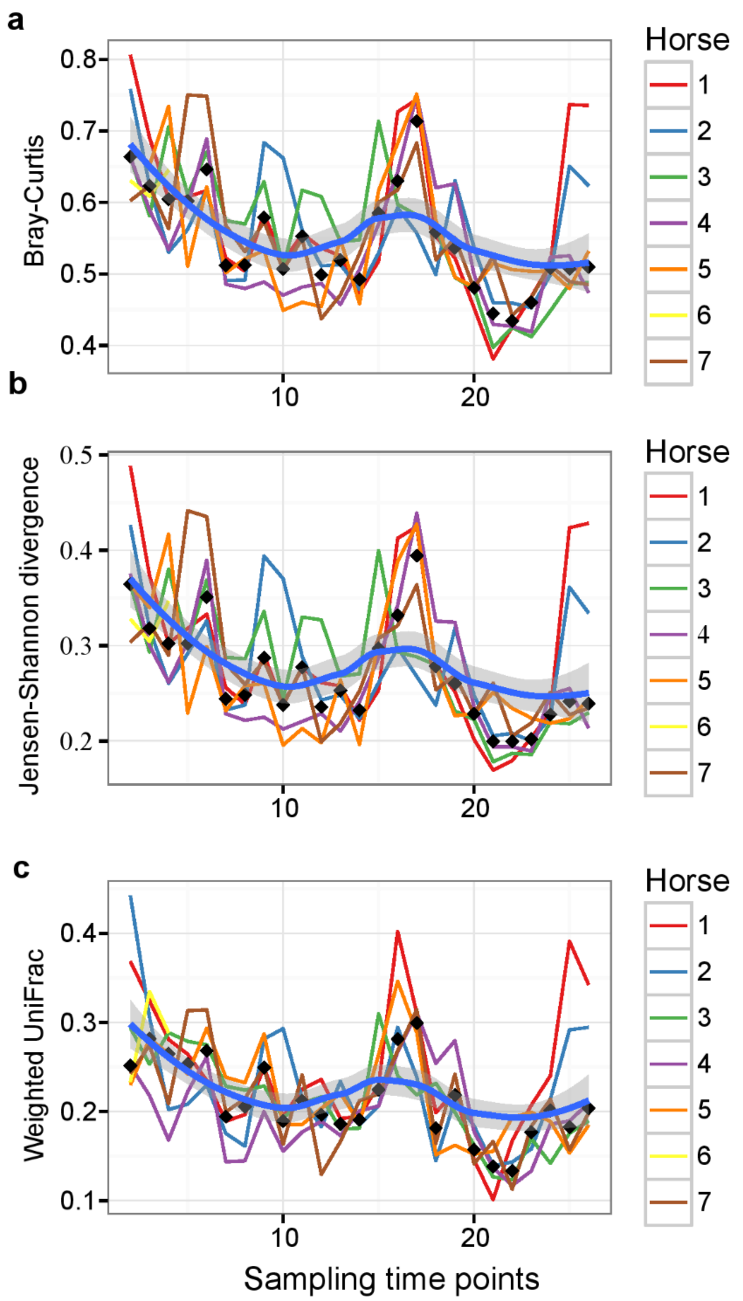

Supplementary Figure S6. Empirical growth plots of beta diversity measures superimposed with loess regression lines. Black diamonds are the median values at each of the sampling time points.

Supplementary Table S1. Demographics of the horses included in the study. The table provides a summary of the age, breed and sex of the 7 horses used in the current study, results of two worm egg counts performed during the study, and the total number of samples collected from each horse. WEC = worm egg count.

**Horse**	**Age (year)**	**Breed**	**Sex**	**First WEC (eggs/g)**		**Second WEC (eggs/g)**		**No. of samples collected**
				WEC	Infection intensity*	WEC	Infection* intensity	
1	4	Warmblood	Gelding	325	Moderate	1125	High	28
2	10	Warmblood	Female	0	Zero	0	Zero	28
3	6	Thoroughbred	Female	825	High	800	High	28
4	4	Welsh Pony	Female	475	High	250	Moderate	28
5	5	Welsh Pony	Female	2000	High	200	Moderate	28
6	3	Warmblood	Gelding	300	Moderate	-	-	5
7	3	Warmblood	Female	575	High	725	High	27

* Infection intensity was classified based on the number of eggs/g of faeces into zero/low infection (<100 eggs/g), moderate infection (100 – 400 eggs/g) and high infection intensity (>400 eggs/g).

Supplementary Table S2. Relative abundance (%) of bacterial phyla identified in the horse faecal microbiota at all sampling time points

Phylum/Time points	T1	T2	T3	T4	T4*	T5	T6	T7	T8	T9	T10	T11	T12	T13	T14	T16	T17
Bacteroidetes	34.49	39.60	35.58	40.29	42.53	43.98	39.23	35.94	40.66	37.51	37.93	45.31	44.41	43.02	48.94	42.78	34.55
Firmicutes	29.89	34.27	33.02	33.81	35.29	26.27	35.55	38.44	34.87	42.71	44.03	34.15	37.65	34.83	26.83	35.30	24.43
Fibrobacteres	14.57	10.31	8.70	4.48	3.45	10.38	7.50	6.03	7.56	4.48	3.55	5.70	4.32	5.53	8.71	6.14	18.58
Spirochaetes	9.21	7.04	11.04	10.94	8.50	10.43	7.80	8.50	7.46	7.08	5.96	6.37	4.34	6.06	7.48	6.56	15.61
Verrucomicrobia	4.30	3.65	5.29	5.17	4.75	4.18	4.51	4.57	2.98	0.99	2.27	2.09	2.62	3.83	2.77	3.84	1.85
Unassigned	2.04	1.25	1.70	1.20	1.31	1.09	1.36	1.66	1.81	2.04	1.85	1.72	1.82	1.94	1.44	1.52	1.30
Proteobacteria	1.97	1.37	1.60	1.64	1.48	1.33	1.46	1.77	1.73	1.60	0.95	1.64	1.30	1.25	1.02	1.21	1.57
Tenericutes	0.47	0.49	0.35	0.36	0.40	0.39	0.34	0.40	0.18	0.42	0.44	0.38	0.62	0.68	0.56	0.37	0.20
Actinobacteria	0.14	0.14	0.18	0.18	0.23	0.18	0.24	0.22	0.20	0.22	0.33	0.19	0.23	0.23	0.23	0.16	0.20
TM7	0.37	0.22	0.23	0.23	0.21	0.13	0.17	0.18	0.12	0.22	0.27	0.17	0.19	0.16	0.10	0.11	0.15
Lentisphaerae	0.15	0.13	0.23	0.13	0.17	0.20	0.22	0.19	0.20	0.09	0.06	0.16	0.12	0.14	0.12	0.14	0.09
Synergistetes	0.04	0.09	0.09	0.10	0.14	0.07	0.10	0.19	0.19	0.24	0.15	0.14	0.22	0.16	0.10	0.20	0.06
Cyanobacteria	0.16	0.08	0.21	0.12	0.10	0.12	0.08	0.13	0.10	0.25	0.12	0.17	0.11	0.06	0.12	0.08	0.06
Armatimonadetes	0.05	0.07	0.04	0.12	0.09	0.09	0.04	0.09	0.10	0.10	0.21	0.08	0.21	0.15	0.11	0.08	0.03
Elusimicrobia	0.03	0.01	0.03	0.02	0.01	0.02	0.01	0.00	0.00	0.01	0.01	0.00	0.02	0.01	0.01	0.00	0.00
SR1	0.06	0.01	0.01	0.01	0.04	0.06	0.01	0.01	0.01	0.00	0.00	0.00	0.00	0.00	0.00	0.01	0.00
Planctomycetes	0.00	0.00	0.00	0.00	0.00	0.01	0.01	0.00	0.00	0.01	0.01	0.01	0.01	0.01	0.01	0.00	0.01
WPS-2	0.01	0.00	0.00	0.00	0.00	0.00	0.00	0.00	0.01	0.00	0.00	0.01	0.00	0.00	0.00	0.00	0.00

Supplementary table S2 continued:

Phylum/Time points	T18	T19	T20	T21	T22	T23	T24	T25	T26	T27	Total (mean)
Bacteroidetes	41.10	36.51	33.13	36.99	34.74	35.31	37.87	35.54	38.97	37.05	39.04
Firmicutes	30.57	30.80	25.70	25.69	26.55	27.34	22.68	28.55	24.22	30.93	31.64
Fibrobacteres	11.36	14.75	15.85	16.63	18.36	17.97	22.99	14.87	14.73	16.06	10.87
Spirochaetes	8.11	7.99	14.71	13.14	13.72	12.64	9.01	11.17	12.96	7.90	9.32
Verrucomicrobia	3.13	4.24	4.58	2.48	1.97	2.00	2.84	3.86	2.07	1.96	3.29
Unassigned	1.89	1.57	1.60	1.30	1.14	1.20	1.42	1.40	2.05	1.46	1.56
Proteobacteria	0.99	1.64	1.68	1.46	1.39	1.27	1.02	2.12	1.76	1.91	1.49
Tenericutes	0.26	0.22	0.30	0.27	0.32	0.33	0.18	0.28	0.33	0.30	0.36
Actinobacteria	0.14	0.20	0.27	0.23	0.23	0.21	0.15	0.25	0.30	0.18	0.21
TM7	0.09	0.12	0.12	0.18	0.10	0.20	0.15	0.24	0.22	0.26	0.18
Lentisphaerae	0.22	0.11	0.15	0.12	0.08	0.10	0.11	0.10	0.16	0.23	0.14
Synergistetes	0.12	0.15	0.13	0.09	0.08	0.08	0.09	0.12	0.07	0.13	0.12
Cyanobacteria	0.01	0.05	0.10	0.07	0.10	0.03	0.02	0.05	0.05	0.08	0.10
Armatimonadetes	0.10	0.08	0.03	0.04	0.05	0.06	0.03	0.03	0.03	0.08	0.08
Elusimicrobia	0.01	0.00	0.06	0.02	0.02	0.03	0.02	0.01	0.01	0.00	0.01
SR1	0.00	0.01	0.01	0.01	0.01	0.01	0.00	0.01	0.01	0.00	0.01
Planctomycetes	0.00	0.00	0.00	0.00	0.00	0.00	0.00	0.00	0.01	0.00	0.00
WPS-2	0.00	0.00	0.00	0.00	0.00	0.00	0.01	0.00	0.01	0.01	0.00

References

1 Turner, S., Pryer, K. M., Miao, V. P. & Palmer, J. D. Investigating deep phylogenetic relationships among cyanobacteria and plastids by small subunit rRNA sequence analysis. *J. Eukaryot. Microbiol.* **46**, 327-338 (1999).

2 Baker, G. C., Smith, J. J. & Cowan, D. A. Review and re-analysis of domain-specific 16S primers. *J. Microbiol. Methods* **55**, 541-555 (2003).

3 Polz, M. F. & Cavanaugh, C. M. Bias in template-to-product ratios in multitemplate PCR. *Appl. Environ. Microbiol.* **64**, 3724-3730 (1998).

4 Caporaso, J. G. *et al.* QIIME allows analysis of high-throughput community sequencing data. *Nat. Methods* **7**, 335-336 (2010).

5 Edgar, R. C., Haas, B. J., Clemente, J. C., Quince, C. & Knight, R. UCHIME improves sensitivity and speed of chimera detection. *Bioinformatics (Oxford, England)* **27**, 2194-2200; 10.1093/bioinformatics/btr381 (2011).

6 DeSantis, T. Z. *et al.* Greengenes, a chimera-checked 16S rRNA gene database and workbench compatible with ARB. *Appl. Environ. Microbiol.* **72**, 5069-5072; 10.1128/aem.03006-05 (2006).

7 Edgar, R. C. Search and clustering orders of magnitude faster than BLAST. *Bioinformatics (Oxford, England)* **26**, 2460-2461; 10.1093/bioinformatics/btq461 (2010).

8 Caporaso, J. G. *et al.* PyNAST: a flexible tool for aligning sequences to a template alignment. *Bioinformatics (Oxford, England)* **26**, 266-267; 10.1093/bioinformatics/btp636 (2010).

9 Wang, Q., Garrity, G. M., Tiedje, J. M. & Cole, J. R. Naive Bayesian classifier for rapid assignment of rRNA sequences into the new bacterial taxonomy. *Appl. Environ. Microbiol.* **73**, 5261-5267 (2007).

10 Price, M. N., Dehal, P. S. & Arkin, A. P. FastTree 2--approximately maximum-likelihood trees for large alignments. *PloS One* **5**, e9490; 10.1371/journal.pone.0009490 (2010).
